# Exploring Efficient and Energy-Saving Microwave Chemical and Material Processes Using Amplitude-Modulated Waves: Pd-Catalyzed Reaction and Ag Nanoparticle Synthesis

**DOI:** 10.3390/molecules30030598

**Published:** 2025-01-28

**Authors:** Satoshi Horikoshi, Tomohiko Mitani, Nick Serpone

**Affiliations:** 1 Department of Materials and Life Sciences, Faculty of Science and Technology, Sophia University, 7-1 Kioi-cho, Chiyoda-ku, Tokyo 102-8554, Japan; 2 Laboratory of Applied Radio Engineering for Humanosphere, Research Institute for Sustainable Humanosphere (RISH), Kyoto University, Gokasho, Uji, Kyoto 611-0011, Japan; mitani@rish.kyoto-u.ac.jp; 3 PhotoGreen Laboratory, Dipartimento di Chimica, Università di Pavia, Via Taramelli 12, 27100 Pavia, Italy

**Keywords:** amplitude-modulated microwaves, energy saving, semiconductor microwave generator, microwave-assisted organic synthesis, Pd-catalyzed Suzuki–Miyaura coupling reaction, nanoparticle synthesis

## Abstract

This study investigated the impact of a 10 kHz amplitude-modulation (AM) wave from a semiconductor microwave generator on the heating of ultrapure water and electrolyte aqueous solutions containing NaCl. It also examined the effects of AM waves on the yields of 4-methylbiphenyl (4-MBP) in the heterogeneous Suzuki–Miyaura coupling reaction, which was conducted in the presence of palladium nanoparticles supported on activated carbon (Pd/AC), as well as their influence on the growth rate during silver nanoparticle synthesis. Applying AM waves, typically used in telecommunications, enhanced heating efficiencies and improved product yields in both the chemical reaction and nanoparticle growth. Irradiating with microwaves under AM conditions allowed it to reduce power output while still achieving target yields and growth rates, even at the same temperatures without AM. This indicates the potential for highly efficient and energy-saving microwave processes in chemical reactions and material synthesis.

## 1. Introduction

An early application of radio waves for heating substances emerged with the concept of “Very Short-Wave Cooking”, introduced in an American magazine for radio experimenters in 1933 [1]. This innovative technology was acclaimed as “the biggest innovation since cooking with fire”. During the Chicago World’s Fair that same year, a demonstration showcasing the cooking of sandwiches and steaks using a 60 MHz VHF transmitter attracted considerable attention. Initial research into using microwaves for chemical reactions includes a patent awarded to Dow Chemical in 1969 for microwave-induced emulsion polymerization of vinyl monomers [2]. Furthermore, a Russian study published in 1973 explored the microwave vulcanization of rubber along with the microwave polymerization of methyl methacrylate and styrene [3]. However, these early investigations failed to generate substantial interest at the time, and the advancement of microwave chemistry appeared to stagnate.

A pivotal moment in the field occurred in 1986 when two influential papers published in Tetrahedron Letters reignited chemists’ interest in this area. Authored by Richard Gedye [4] and Raymond J. Giguere [5], these papers explored the application of microwave ovens in organic syntheses. Gedye and his team conducted 14 distinct organic syntheses using a Japanese microwave oven and a closed Teflon container. Their findings demonstrated that reaction rates could be accelerated by as much as 240 times—and sometimes even up to 1000 times—compared to traditional oil bath heating. Simultaneously, Giguere and his colleagues observed rapid heating significantly enhanced the synthesis rate. These studies underscored essential factors in microwave chemistry, such as the significance of reaction containers, solvents, temperature control, and the effects of overheating. They also identified challenges and presented new avenues for exploration to many researchers.

In recent years, the chemical and materials industries have experienced significant transformation. Before the Industrial Revolution, human activities largely depended on solar energy; however, with the introduction of coal, there was a shift towards harnessing chemical energy, facilitating the transition from agricultural to industrial economies. The increasing reliance on fossil resources subsequently spurred the growth of the chemical and materials sectors, which heavily depended on these resources to meet the considerable energy demands of their processes. Nonetheless, this dependence on fossil fuels has resulted in severe environmental degradation and resource depletion, prompting a widespread call for sustainable development. Currently, efforts towards decarbonizing these industries are progressing, aiming for carbon neutrality and necessitating substantial changes [6]. In this context, microwave heating is emerging as a promising solution, offering enhanced energy efficiency and significant reductions in CO_2_ emissions in chemical and materials processes, particularly in industrial applications. Several microwave-based facilities have already been established, and future advancements in microwave power technology are crucial for further improving energy savings [7].

The first experiment that utilized amplitude-modulated waves occurred in 1906 when a voice message was transmitted from Brant Rock, Massachusetts, and successfully received by a ship hundreds of miles away [8]. Since then, amplitude-modulated waves have been used for television video distribution and satellite broadcasting. Today, amplitude modulation is a widely utilized technique in electronic communications for transmitting information via radio carrier waves, so it is a mature technology. On the other hand, there are very few cases where AM waves are incorporated into the use of microwaves as an energy source. This is just an example of investigating the behavior of microwave plasma generation using AM (micro)waves [9]. Conversely, a detailed literature search revealed no examples of amplitude-modulated waves used in heating. Remarkably, more than a century later, this established technology in the field of communications is herein being explored for the first time for its potential use in the heating field, especially as it would be of significant interest in microwave chemistry.

To the best of our knowledge, heating experiments using amplitude-modulated waves in microwave chemistry are practically non-existent, and thus, the study presents a range of cases that is as varied as possible. In particular, chemical yields in syntheses in the presence of solid catalysts cannot be discussed based on heating temperature, not to mention that catalyst deactivation by microwave discharges can also affect the yields, hence the interest in exploring how amplitude modulation affects the three cases noted below and, in particular, how the 10 kHz tempo caused by this modulation might affect the morphology of nanoparticles.

Consequently, the present study was undertaken to look at the effects of amplitude-modulated waves in three individual cases. First, we explore the benefits of employing amplitude-modulated (AM) waves for enhancing heating in ultrapure water and in electrolyte (NaCl) aqueous solutions. Second, we highlight the advantages of amplitude-modulated waves in synthesizing 4-methyl biphenyl (4-MBP) through the Suzuki–Miyaura coupling reaction in a palladium-catalytic heterogeneous organic medium. Third, we examine how amplitude-modulated waves affect the growth rate of silver nanoparticles as an example of the synthesis of nanomaterials.

## 2. Results and Discussion

### 2.1. Microwave Heating of Ultrapure Water and an Electrolyte Aqueous Solution

**Figure 1:**
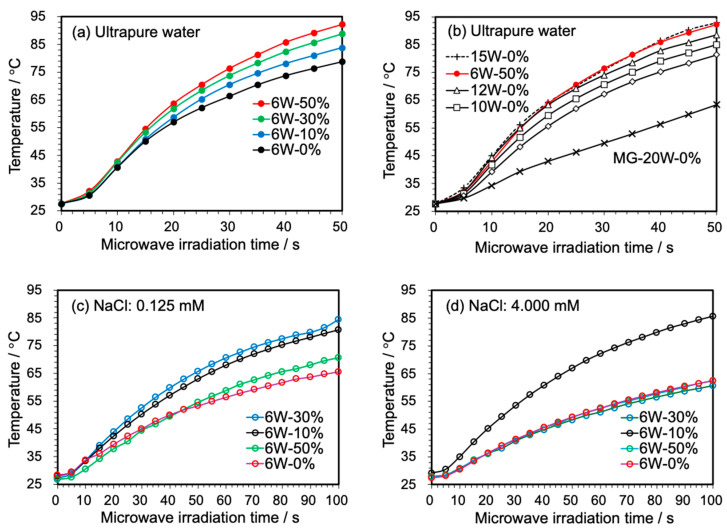
Temperature–time profiles in the heating of (**a**,**b**) ultrapure water and (**c**,**d**) NaCl aqueous solution (each 0.5 mL) placed in a quartz sample tube and subjected to irradiation with 6 W microwaves (2.45 GHz) generated from a semiconductor generator with and without 10 kHz amplitude modulation (amplitude-modulated wave) and 20.0 W microwaves from a conventional magnetron generator. Description: 6W-0%: 6.00 W power with no modulation; 6W-10% to 6W-50%: 6.00 W power with 10–50% modulation with 10 kHz amplitude modulation; 10W-0% to 15W-0%: 10–15 W power with no modulation; MG: magnetron, 20.0 W power with no modulation.

The increase in water temperature from ultrapure water samples exposed to microwave irradiation (with an input power of 6.00 W) for up to 50 s is depicted in Figure 1a. This experiment utilized a single-mode applicator paired with a semiconductor generator, both with and without amplitude modulation (AM) set at 10 kHz. Without the amplitude-modulated wave, the water temperature reached 78.8 °C after 50 s of irradiation with 2.45000 GHz microwaves. In contrast, incorporating the 10 kHz amplitude-modulated wave markedly enhanced heating efficiency, yielding water temperatures of 83.8 ± 0.3 °C at 10% modulation, 88.8 ± 0.2 °C at 30% modulation, and 92.2 ± 0.4 °C at 50% modulation.

Due to the characteristics of amplitude-modulated waves, a microwave power level exceeding the designated setting was utilized to irradiate the sample [10]. To quantify the microwave power employed for irradiation when the semiconductor oscillator was set to 6.00 W, we used an Agilent Technologies EXA signal analyzer N9010A. The 10%, 30%, and 50% modulation levels adjusted the power to 6.03 W, 6.26 W, and 6.67 W, respectively. When using a single sine wave for modulation, it is essential to recognize that if power is considered the reference standard, even with a modulation depth of 1.00 (100%), the power allocated to both sidebands is only 0.50 (½) of the totals. In contrast, for a single sideband, it is further reduced to 0.25 (¼). The increase in microwave power associated with the amplitude-modulated wave can be explained using Equation (1) [10]: *P_AM_* denotes the total power of the amplitude-modulated wave, *P_c_* indicates the power in a continuous wave, and *m* represents the modulation depth, which is constrained within the range of 0 (0%) ≤ m ≤ 1 (100%).

(1) $$P_{AM}=P_{C}\left(1+\frac{m^{2}}{2}\right)$$

The increase in water temperature may be attributed to the rise in microwave power associated with the amplitude-modulated (AM) wave. However, can a modest increase in power output genuinely result in such a significant change in water temperature? To investigate this question, we examined the effects of microwave irradiation on a water sample at power levels of 8.00, 10.0, 12.0, and 15.0 W, applied for a duration of 50 s without the amplitude-modulated wave from the semiconductor generator. The measured temperatures were 81.3 °C at 8.00 W, 84.9 °C at 10.0 W, 88.5 °C at 12.0 W, and 92.8 °C at 15.0 W (see Figure 1b). Our results indicate that an increase in microwave power corresponds to a more rapid rise in water temperature. Notably, even when the microwave input power was doubled from 6.00 W to 12.0 W, the heating efficiency remained lower than the temperature achieved with the amplitude-modulated wave at 50% modulation at 6.00 W. It was only at a power level of 15.0 W that the heating rate began to align. In conclusion, the effectual microwave power output using the amplitude-modulated wave at 50% modulation is 6.67 W, while achieving a comparable heating rate with the non-amplitude-modulated wave requires a power of 15.0 W, resulting in energy savings of approximately 56%.

We anticipate that modulation will affect the relaxation process as the rotation of water clusters (or individual water molecules) becomes increasingly irregular under modulation. Furthermore, we expect to observe a synergistic effect from the interaction of 2.45 GHz microwaves with water orientation when subjected to 10 kHz modulation. It is important to note that the dielectric relaxation of water pertains to the speed at which molecular orientation can align with the electric field, typically exhibiting a Debye-type relaxation time of approximately 8 ps [11]. In contrast, the period of 10 kHz spans about 100 µs, which is significantly longer than the relaxation time. Additionally, the energy relaxation time of the OH stretching vibration mode in water clusters is around 50 fs [12], highlighting a substantial discrepancy as well.

**Figure 2:**
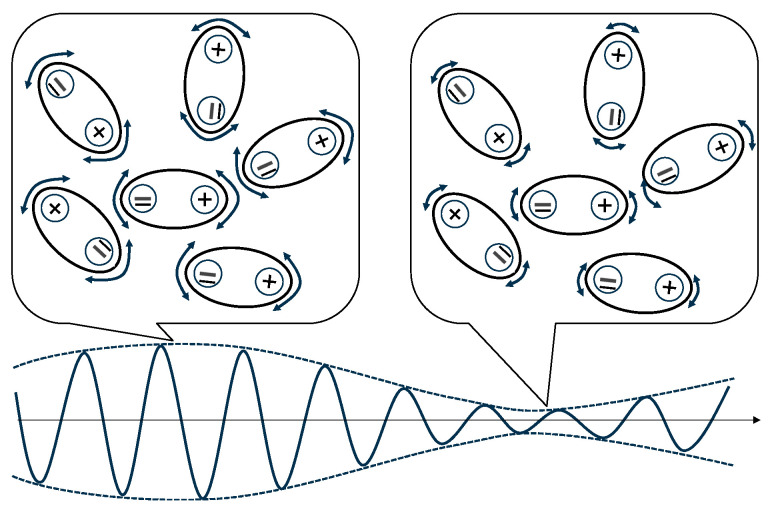
Image of molecular motion under AM wave irradiation.

The differences observed may be attributed to an indirect effect. Unlike continuous waves (CWs), which deliver energy at a steady intensity, microwaves with amplitude modulation (AM) vary their energy output over time. This fluctuation impacts both the timing and the quantity of energy absorbed by water molecules (see Figure 2). Notably, the maximum absorption rate of water within the microwave spectrum occurs around 10 GHz, while 2.45 GHz is not optimal for absorption [13]. Therefore, utilizing a low-frequency amplitude-modulated wave, such as 10 kHz AM, could result in nonlinear behavior in energy delivery, and the resultant localized temperature variations in the water may indirectly affect the relaxation time.

The behavior of microwave heating in an aqueous electrolyte solution is fascinating. Generally, the mechanism by which a material absorbs microwaves and generates heat involves the three components outlined in Equation (2) [14]. The first term represents conduction loss heating, the second term indicates dielectric loss heating, and the third term accounts for magnetic loss heating. It is important to note that *E* and *H* denote the strengths of the electric and magnetic fields of the microwaves, respectively; *f* represents the frequency of the microwaves; *ε*_0_ is the dielectric constant in a vacuum; *ε*_r_″ refers to the relative dielectric loss factor; *μ*_0_ signifies the magnetic permeability in a vacuum; and *μ*_r_″ denotes the relative magnetic loss.

(2) $$P=\frac{1}{2}\sigma {\left|E\right|}^{2}+\pi f\varepsilon_{0}\varepsilon_{r}\prime \prime {\left|E\right|}^{2}+\pi f\mu_{0}\mu_{r}\prime \prime {\left|H\right|}^{2}$$

In the case of ultrapure water, heating occurs primarily through the second term of Equation (2), which represents dielectric loss heating. In contrast, for electrolyte solutions, heating is driven by the first term, related to conduction loss heating, and the second term, associated with dielectric loss heating. This raises the question: what impact does the amplitude-modulated wave have on conduction loss heating? The results illustrated in Figure 1c,d demonstrate that when heating an electrolyte solution containing 0.125 mM NaCl, the heating efficiency improves as the modulation rate increases to 10% and 30%, similarly to what is observed with ultrapure water when microwave heating incorporates AM. However, at a modulation rate of 50%, there is a significant drop in heating efficiency, and the influence of the amplitude-modulated waves is nearly diminished. A previous study [15] indicated that an electrolyte solution’s microwave absorption rate increased due to dielectric and conduction loss heating, reducing the microwave penetration depth and leading to slower microwave heating efficiency. This explains why the heating rates shown in Figure 1c,d are slower than those in Figure 1a.

In contrast, when the concentration of NaCl is set at 4.000 mM, the effect of amplitude modulation (AM) is observed in only 10% of cases, with the remainder showing no effect. This raises the question of why certain concentrations align with specific modulation rates to enhance the heating rate. At a lower concentration of 0.125 mM, ionic interactions are anticipated to increase, leading to a somewhat delayed response to changes in the electric field and complex absorption characteristics [16]. Conversely, at the higher concentration of 4.000 mM, the response to the electric field is expected to be constrained due to intense interactions among the ions and the formation of clusters. This subsequently results in significant alterations in energy dissipation and dielectric properties at a modulation frequency of 10 kHz.

In essence, ions in an aqueous solution exhibit random Brownian motion; when an external electric field is applied, they experience a directional drift motion. However, the typical relaxation times for Na^+^ and Cl^−^ are in the range of a few nanoseconds (ns) [17], allowing them to quickly respond to electric field variations at 10 kHz (with a period of 100 μs), suggesting that this alone may not be the direct cause. At present, the underlying principle remains inadequately defined. Nevertheless, we propose the following hypothesis: Na^+^ and Cl^−^ are strongly associated with water molecules, forming a hydration shell. The 10 kHz amplitude-modulated wave may act as an external stimulus, occurring at a frequency close to the relaxation time of the hydration shell around the ions. This interaction could enhance the activity of the ions and, in turn, improve heating efficiency. This effect seems most pronounced when the concentration of ions aligns with the degree of modulation.

Next, we explored the heating of ultrapure water using a 2.45 GHz magnetron microwave generator that lacked amplitude-modulated waves (refer to Figure 1b). The heating rate of the water produced by the magnetron generator, which emitted three times more potent microwaves (20 W) than those from a 6 W semiconductor generator, was notably slower, reaching only 63.3 °C after 50 s. This suggests that when comparing a semiconductor generator utilizing 50% AM to a magnetron generator, the heating efficiency of the semiconductor generator can be increased by 1.8 times, even with a 70% reduction in power consumption. This raises the question: why are magnetrons so inefficient? We analyzed the microwave spectrum produced by these various microwave generators to address this issue.

**Figure 3:**
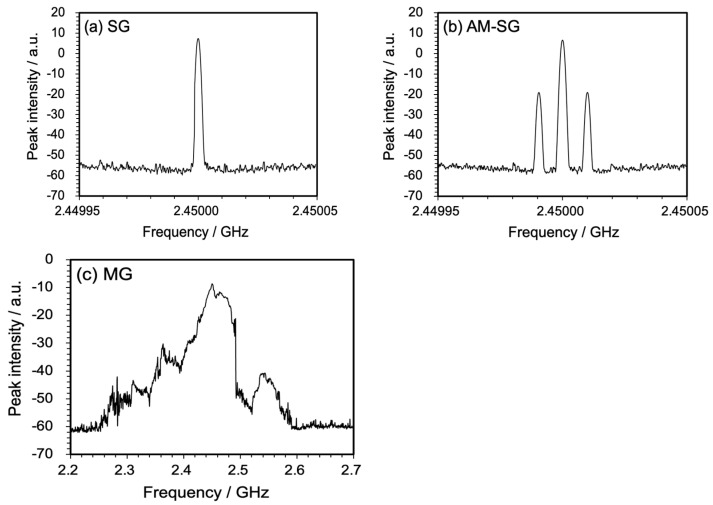
Spectra of the 2.45 GHz microwave irradiation from (**a**) the semiconductor generator (SG), (**b**) the semiconductor generator with a 10 kHz–10% amplitude-modulated wave (AM-SG), and (**c**) the magnetron generator (MG).

The semiconductor generator produced microwaves within a narrow frequency range of 2.45000 ± 0.00250 GHz (Figure 3a). The microwave power was primarily centered around the 2.45000 GHz frequency, allowing resonance in the single-mode apparatus. The spectrum at this 2.45000 GHz frequency, to which a 10 kHz, 10% amplitude-modulated wave was applied, is illustrated in Figure 3b. Within this frequency domain, the amplitude-modulated wave generated a signal with power focused on the primary frequency of 2.45000 GHz, alongside two adjacent sidebands situated at 2.4499905 GHz and 2.4500105 GHz. Each of these sidebands shares the same bandwidth as the modulated signal and acts as a mirror image of one another. Notably, as the gain gradually increased from 10% to 30% and eventually to 50%, the intensity of the 2.45000 GHz peak remained constant, while the intensities of the sideband peak at 2.4499905 and 2.4500105 GHz increased. These three peaks exhibit minimal frequency difference, facilitating resonance within a single-mode applicator. In contrast, the frequency distribution of microwave radiation from the magnetron generator (shown in Figure 3c) covered a broader frequency range of 2.25–2.60 GHz, which varied based on the specific characteristics of the magnetron microwave generator. Consequently, the actual output of 2.45 GHz microwaves was lower than the input power, resulting in these microwaves being unable to resonate within the single-mode apparatus coupled with a magnetron.

### 2.2. Product Yields of 4-Methylbiphenyl (4-MBP) from the Suzuki–Miyaura Coupling Reaction

Given the demonstrated advantages of enhanced water heating efficiency through microwave heating under amplitude-modulation (AM) conditions, an investigation was conducted to evaluate the impact of amplitude-modulated waves on organic synthesis using a solid catalyst. The yields of 4-MBP produced from the Suzuki–Miyaura coupling reaction (chosen as a model reaction) after a 20-minute irradiation period under various microwave conditions are summarized in Table 1. Under non-amplitude-modulated wave conditions, the chemical yield of 4-MBP was 11.3 ± 1.0% at a power output of 15.0 W. Preliminary experiments had indicated a lower yield at 5.0 W, even after 20 min of synthesis, when the microwaves operated at lower power settings of 10.0 W or below. Therefore, the output was set at 15.0 W to minimize significant discrepancies in evaluating the differences among the conditions.

Under conditions of amplitude-modulated waves, the yield notably increased to 16.0 ± 1.0% at 7.50 W (10 kHz, 50% AM). It is important to highlight that with 7.50 W of microwave energy and the specified modulation settings, the theoretical microwave power irradiating the sample was calculated to be 8.44 W (as detailed in Equation (1)). Furthermore, under AM conditions, with a power setting of 15.0 W, the yield rose to 18.1 ± 1.0% (15.0 W; 10 kHz, 50% AM), where the effective irradiation power on the sample was determined to be 16.88 W. The variations in product yields can be attributed to the modulation of the microwaves. Specifically, microwaves operating at 7.50 W with 50% AM at 10 kHz consume half the power compared to those running at 15.0 W without AM, yet they achieve approximately a 1.4-fold increase in yield. Additionally, using amplitude-modulated waves at a power consumption of 15.0 W led to enhanced yields up to 1.6 times at equivalent power levels. It is essential to note that these experimental yields represent the average results from three separate experiments.

During the experiment, careful data observation revealed that in the absence of an amplitude-modulated wave at 15.0 W, bubbles began to form on the catalyst surface after approximately 114 s. In contrast, when an amplitude-modulated wave was employed at 7.50 W, bubbles were observed after roughly 71 s. In the experiment with the amplitude-modulated wave at 15.0 W, they appeared after about 41 s. This difference can be attributed to the selective heating of the Pd/AC catalyst by microwaves, which subsequently heats and vaporizes the nearby solvent. The results demonstrated that using an amplitude-modulated wave significantly enhanced heating efficiency. Following this, refluxing was maintained at around 110 °C. Previous studies indicated that irradiation with pulsed microwaves, which were alternately turned on and off, minimized micro-discharges on the surface of the Pd/AC catalyst, ultimately extending the latter’s lifespan [18]. Observations made with a high-speed camera confirmed that discharges occurred even when using the amplitude-modulated wave; however, the frequency of these discharges was found to be reduced. Clearly, the advantages of the amplitude-modulated wave extend beyond an increased heating rate, as it also contributes to decreased catalyst deterioration caused by microwave discharges. Conversely, it is possible that the periodic changes induced by the amplitude-modulated wave could have influenced the contact efficiency between the substrate and the Pd-catalytic surface.

### 2.3. Synthesis of Ag Nanoparticles (Ag-NPs)

**Figure 4:**
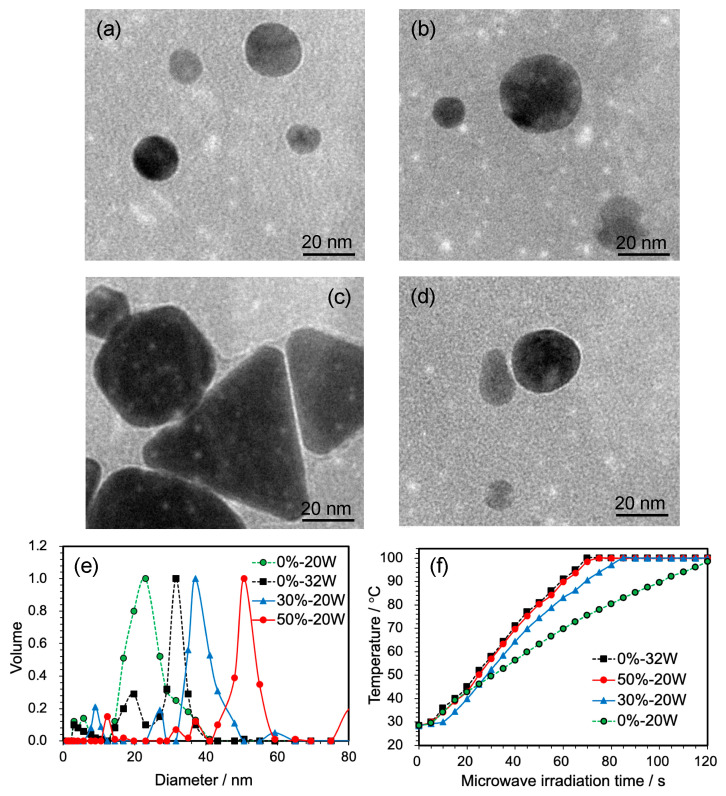
TEM images of the Au nanoparticles (Ag-NPs) produced by 120 s irradiation of (**a**) 20 W-microwave without amplitude-modulated wave, (**b**) 20 W-microwave with amplitude-modulated wave (10 kHz, 30%), (**c**) 32 W microwave without amplitude-modulated wave and (**d**) 20 W microwave with amplitude-modulated wave (10 kHz, 50%). (**e**) Ag nanoparticles’ size distribution with a dynamic light scattering measurement produced by microwave with or without amplitude-modulated wave condition. (**f**) Temperature–time profiles in the heating of Ag-NPs under varied microwave irradiation.

The growth and characteristics of amplitude modulation (AM) microwaves on silver nanoparticles (Ag-NPs) were explored within the framework of nanomaterial synthesis. For the preliminary investigation, a microwave output of 18 W or higher was required to raise the reaction solution’s temperature to at least 100 °C; thus, this experiment employed an output setting of 20 W. Without AM (0%-20 W), spherical Ag-NPs were synthesized (see Figure 4a), with dynamic light scattering (DLS) revealing the most prevalent particle size to be 23.1 nm (Figure 4e). However, there was a relatively wide distribution of sizes. In contrast, spherical particles remained evident when Ag-NPs were produced under 30% AM (10 kHz, 20 W) (Figure 4b). Still, the predominant particle size increased to 37.0 nm (Figure 4e), and the size distribution became narrower. Furthermore, under 50% AM (10 kHz, 20 W), the synthesis resulted in not only spherical particles but also triangular (and some hexagonal) shapes (Figure 4c). The primary particle size experienced a significant increase to 50.7 nm (Figure 4e), along with a narrower size distribution.

Next, the changes in synthesized particles were analyzed from the perspective of reaction temperature. Figure 4f demonstrates the temperature variations observed under each microwave condition. When microwave irradiation was 0–20 W, the solution temperature reached 100 °C in approximately 120 s. In contrast, under the 30% amplitude-modulated wave (10 kHz, 20 W), the temperature reached 100 °C in about 85 s. With the 50% amplitude-modulated wave (10 kHz, 20 W), the same temperature was reached within about 70 s. This suggests that the rapid heating effect of the amplitude-modulated wave facilitated an enhanced growth of particle size in Ag-NPs.

Replicating the temperature profile achieved with the 50% amplitude-modulated wave (10 kHz, 20 W) using microwave irradiation without the modulation (0%) necessitated an increase in microwave power. Specifically, a microwave power of 32 W was required to reproduce the temperature profile observed under the 50% amplitude-modulated wave (10 kHz, 20 W), and the resulting profile was similar (see Figure 4f). When silver nanoparticles (Ag NPs) were synthesized under this heating condition, the resulting particles were smaller compared to the spherical particles produced under the amplitude-modulated wave conditions (refer to Figure 4d). Dynamic light scattering (DLS) revealed that the most prevalent size was 31.62 nm. Therefore, synthesizing Ag NPs through microwave irradiation under amplitude-modulated wave conditions can reduce power consumption by approximately 38%, while also enhancing the particle growth rate by a factor of 1.6.

## 3. Materials and Methods

### 3.1. Microwave Setup

**Figure 5:**
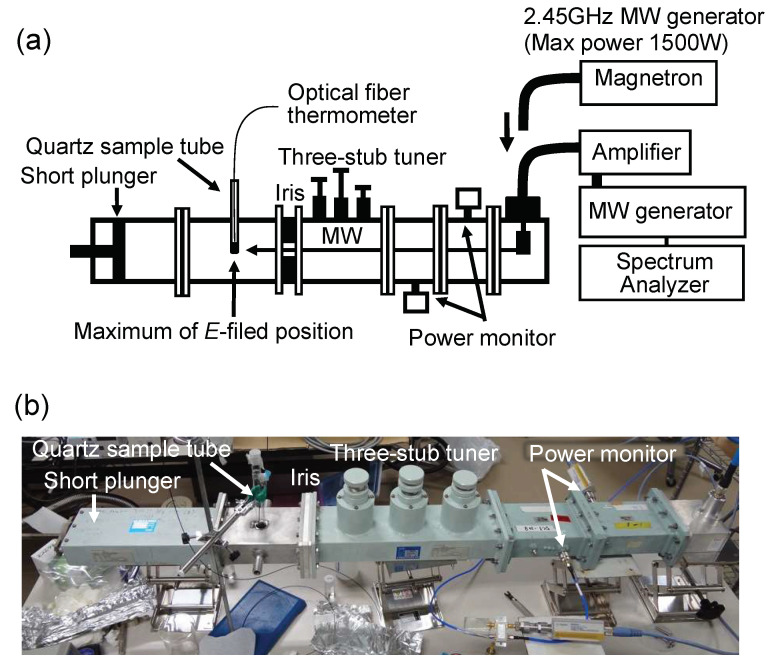
Details: (**a**) image and (**b**) picture of the experimental setup in the single-mode microwave applicator.

The continuous microwave irradiation setup featuring the single-mode cavity TE_103_ (transverse electric 103 mode) utilized in our experiments is schematically depicted in Figure 5a,b. Ultrapure water (Nomura Micro Science Co., Ltd., Kanagawa, Japan; semiconductor cleaning water; 18 MΩ cm^−1^; 1.0 mL) or NaCl aqueous solution (0.125 mM and 4.000 mM) was introduced into a quartz sample tube reactor with an internal diameter of 4.0 mm. This reactor was positioned within the waveguide at maximal microwave electric field (E-field) density. The wavelength of the microwaves propagating in the TE_103_ mode within the waveguide was approximately 14.7 cm, as determined in previous studies [19]. The peak density of the electric field occurred at three-quarters of the wavelength of the standing wave in the waveguide, specifically at 11.03 cm.

The positioning of the maximum electric field for the sample was fine-tuned using a short plunger, a three-stub tuner, an iris, and a power monitor. We employed an automatic short plunger with an Agilent Technologies 8720C Network Analyzer to minimize microwave reflections within the setup. The semiconductor microwave generator was an Agilent Technologies MXG system with an irradiation range from 100 kHz to 32 GHz. The microwaves produced by the semiconductor generator were amplified using a GA0827-4754-R power amplifier (R&K Co., Ltd., Fuji-City, Shizuoka-Pref., Japan) and transmitted into the waveguide via a coaxial cable. Additionally, a conventional magnetron microwave generator (Kajimoto Ceramics Co., Saitama, Japan; PMBGT magnetron; 2.45 GHz; maximum power of 1500 W) was utilized for comparative purposes to assess its impact on heating the water sample. For the magnetron system, a coaxial waveguide converter was installed at the magnetron’s waveguide position and linked to a coaxial cable, similar to the configuration of the semiconductor microwave generator. The temperature of the water sample was monitored using an optical fiber thermometer (FL-2000, Anritsu Meter Co., Ltd., Tokyo, Japan). The frequency distributions of the irradiating microwaves were observed with an Agilent Technologies EXA signal analyzer.

**Figure 6:**
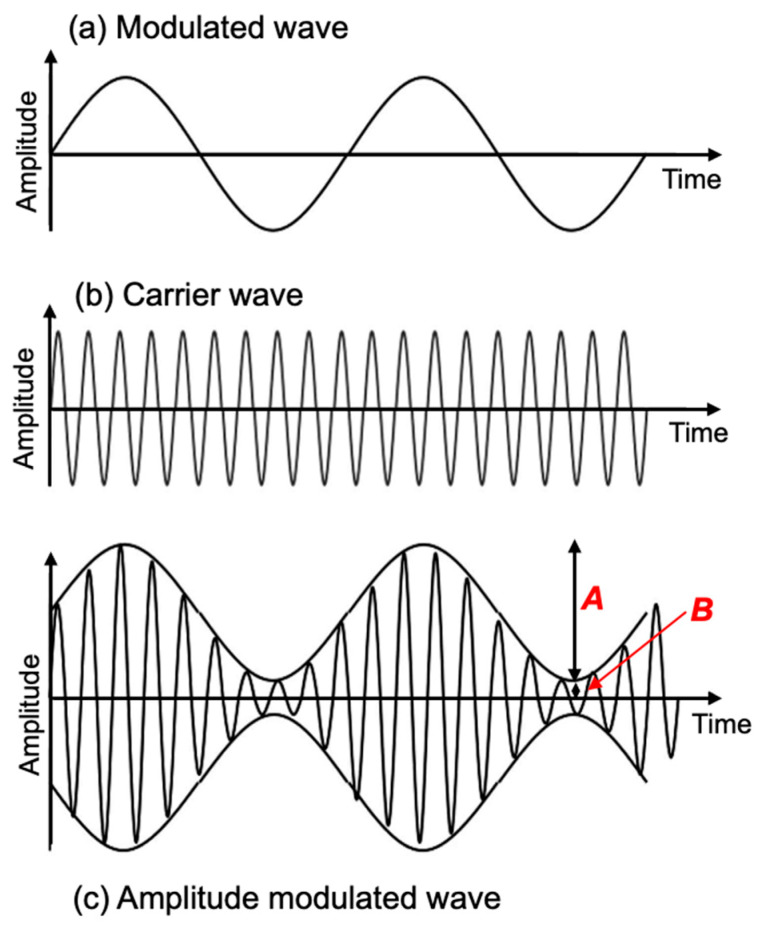
Configuration image of an amplitude modulation (amplitude-modulated wave) of the 2.45 GHz microwaves: (**a**) modulated wave, (**b**) carrier wave, and (**c**) amplitude-modulated wave. The ***A*** and ***B*** in (**c**) denote the maximal and minimal amplitudes.

The 10 kHz amplitude modulation (AM) for the 2.45 GHz microwaves was set to intensities of 10%, 30%, and 50%. The spectral images of the amplitude-modulated wave are illustrated in Figure 6. These modulated waves are produced by modulating and synthesizing the modulated wave (Figure 6a) and the carrier wave at 2.45 GHz (Figure 6b) using the AM function of the microwave generator. The amplitude of the generated modulated wave (Figure 6c) fluctuates. When the ratio of ***A*** to ***B*** in Figure 6c is, for instance, 1:1, the intensity reaches 50%—a lower intensity ratio results in a smaller difference between the minimum and maximum amplitudes. Therefore, as the proportion of ***A*** decreases, the percentage value also diminishes. It is important to note that amplitude-modulated waves do not affect the microwave frequency, which remains at 2.45 GHz in this case.

### 3.2. Synthesis of 4-Methylbiphenyl (4-MBP)

The Pd/AC catalyst was synthesized following the methodology outlined in previous research [20]. Activated carbon particles (AC; 5.0 g; mesh size of 0.95 mm) were initially washed with ultrapure water and a NaOH solution (2.0 M, 50 mL) for 24 h at room temperature. After this treatment, the AC was rinsed with ultrapure water and dried at 100 °C for several hours. Following drying, 1.0 g of the washed AC was placed in an aqueous solution containing PdCl_2_ (0.034 g) and HCl (1.0 M, 50 mL). The pH of the solution was adjusted to 14 by adding NaOH. NaBH_4_ (0.016 M) was then introduced to the solution and stirred for 3 h, followed by an additional stirring period of 2 h at 60 °C. Ultimately, the colloidal Pd/AC particles were filtered, washed with ultrapure water, and dried overnight at 100 °C. The amount of Pd on the activated carbon support, with an approximate diameter of 0.65 mm, was determined to be around 1.6 wt.% using atomic emission spectroscopy with a Shimadzu ICPE-9000 apparatus.

**Scheme 1:**
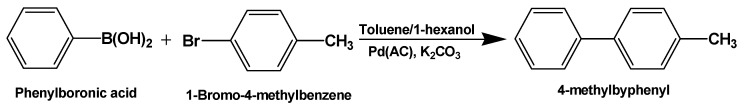
Synthesis of 4-methylbiphenyl (4-MBP) from phenylboronic acid and 1-bromo-4-methylbenzene by the Suzuki–Miyaura cross-coupling process in the presence of the Pd/AC catalyst and K_2_CO_3_ base in a 1-hexanol/toluene solvent system.

The synthesis of 4-MBP through the Suzuki–Miyaura cross-coupling reaction (Scheme 1) was inspired by a prior study [20]. The reaction setup involved palladium deposited on activated carbon (Pd/AC) catalyst particulates (0.18 g), phenylboronic acid (0.96 mmol; 0.12 g), 1-bromo-4-methylbenzene (0.72 mmol; 0.12 g), and potassium carbonate (K_2_CO_3_) as the base (1.4 mmol; 0.20 g). These components were combined with a mixed solvent of toluene and 1-hexanol (6 mL; volume ratio of 1:1) and transferred to a quartz sample tube (internal diameter: 17 mm) under an argon atmosphere. A small condenser was attached to the top of the reactor, and the resulting solution was subjected to microwave irradiation while stirring.

The yields of 4-MBP were determined using gas chromatography with a Shimadzu model 2014 apparatus, which featured a Shimadzu GLC Ultra alloy-1 capillary column. Helium served as a carrier gas, with a column temperature between 100 and 260 °C and a heating rate of 20 °C per minute. A pure sample of 4-MBP (obtained from Fujifilm Wako Pure Chemical Industries, 100% GC standard) was utilized for calibration via gas chromatography. Product yields were calculated through quantification based on the calibration curve.

### 3.3. Synthesis of Silver Nanoparticles (Ag-NPs)

The synthesis of silver nanoparticles (Ag-NPs) was conducted as a model reaction under various microwave conditions, building upon findings from a previous study [21]. A high-purity grade of AgNO_3_ (Kanto Chemical Co., Inc., Tokyo, Japan; 3.14 g), 3 mL of aqueous NH_3_ (28% concentration, Kanto Chemical Co., Inc., Tokyo, Japan), and 10 mL of ultrapure H_2_O were utilized to prepare the diamine-silver (I) complex. Subsequently, a 40 mL solution (60 mM) of [Ag(NH_3_)_2_]^+^ was added to an aqueous solution of carboxymethylcellulose (CMC; 0.05% *w*/*v*; volume of CMC solution: 40 mL). Ten milliliters of the resulting solution were transferred to a quartz sample tube (internal diameter: 17 mm). A small condenser was attached to the top of the reactor, and the mixture was subjected to microwave irradiation while being stirred. The morphologies and size distributions of the synthesized silver nanoparticles were analyzed using a transmission electron microscope (TEM) with a Hitachi High-Technologies Co. (Tokyo, Japan) H-7650 electron microscope. Particle size distributions were assessed via dynamic light scattering (DLS) techniques using an Otsuka Electronics Co., Ltd. (Tokyo, Japan) DLS-8000 series apparatus.

## 4. Concluding Remarks

Microwave heating has long been acknowledged for its potential energy-saving benefits compared to traditional heating methods. This research has confirmed that it conserves energy and reduces heating times by accelerating the heating rate through the microwaves’ amplitude modulation (AM). Efficient temperature rise can be achieved even at low microwave power (energy-saving conditions) by devising a microwave irradiation method, a phenomenon of significant interest to microwave chemistry. Our findings indicate that the behavior of electrolyte solutions is more complex and, in some instances, does not appear to benefit from amplitude-modulated waves. Importantly, utilizing amplitude modulation of microwaves—often applied in communications—enhances energy efficiency and improves chemical yields in organic synthesis when solid catalysts are used. For instance, results show that the yield of 4-MBP is higher despite the lower power consumption when using amplitude-modulated waves. Moreover, the study has demonstrated that low-power applications can also foster an increased growth rate of (silver) nanoparticles during the synthesis (again, energy savings). This effect appears to arise from the interplay between the modulation tempo and the microwave-induced waves, which create a synergistic impact on molecules, thereby accelerating chemical reactions and microwave material processing. Consequently, a deeper understanding of the interactions between amplitude modulation and molecular behavior will be a significant advantage in Microwave Chemistry and Materials Processing, which can pave the way toward innovative industrial applications in these two areas.

## Figures and Tables

**Table 1:** Product yields of 4-methyl biphenyl (4-MBP) at 110 °C in toluene solvent with and without microwave and amplitude-modulated wave conditions under irradiation for 30 min.

AM	Product Yield of 4-MBP/%	Setup Value of Microwave Power/W	Theoretical Microwave Power Irradiated to the Sample/W
None	11.3 ± 1.0	15.0	15.00
10 kHz–50%	16.0 ± 1.0	7.50	8.44
10 kHz–50%	18.1 ± 1.0	15.0	16.88

## Data Availability

Data are contained within the article.
